# Applying Resilience Promotion Training Among Special Forces Police Officers

**DOI:** 10.1177/2158244015590446

**Published:** 2015

**Authors:** Judith P. Andersen, Konstantinos Papazoglou, Mari Koskelainen, Markku Nyman, Harri Gustafsberg, Bengt B. Arnetz

**Affiliations:** 1 University of Toronto Mississauga, Ontario, Canada; 2 Police University College, Tampere, Finland; 3 Wayne State University, Detroit, MI, USA

**Keywords:** police special forces, resilience promotion, critical incidents, physiological reactivity

## Abstract

Police Special Forces (a.k.a. special weapons and tactics [SWAT]) officers are tasked with responding to the most critical situations, including incidents that require specialized skills and equipment beyond typical policing activities. In this study, we tested the feasibility of applying Arnetz and colleagues’ resilience promotion training that was developed for patrol officers to SWAT team officers (*n* = 18). The resilience promotion training program included psychoeducation focused on police stress and resilience, and the practice of resilience promotion techniques (controlled breathing and imagery) while listening to audio-recorded critical incident scenarios. The aims of this study were to (a) examine if a resilience training program was relevant and accepted by SWAT team officers and (b) assess participants’ physiological stress responses (heart rate, respiration) during the resilience training sessions to note if there were improvements in stress responding over time. Our findings revealed that participants were able to significantly reduce their average heart rate and improve their ability to engage in controlled respiration (i.e., breathing) during simulated critical incidents over the course of the 5-day training. Improvements in stress responding were observed even when the critical incident scenarios became more graphic. Results suggest that an intervention to reduce stress responses of SWAT officers to critical incident scenarios works in a simulated training setting. Translation of these findings to real-world occupational hazards is a recommended next step.

## Introduction

Police officers encounter multiple potentially traumatic incidents and extremely stressful situations (e.g., violent criminals, loss of a partner while on duty) as part of their work. Special Forces Police (a.k.a. SWAT [special weapons and tactics]) are tasked with responding to the most critical situations, including incidents that require specialized skills and equipment beyond typical policing activities (Scanff & Taugis, 2002). For example, as reviewed by Ollhoff (2012), SWAT teams are activated in hostage and terrorist situations, active shooter incidents (e.g., school shootings), and to capture dangerous criminals involved in drug and gang activities among many other high risk duties. SWAT officers are involved in long missions that often turn into high pressure critical incidents that require life and death decisions to be made extremely quickly. Some research indicates that due to selection criteria, police officers, and particularly, SWAT officers may be generally more resilient than the average civilian (Galatzer-Levy, Madan, Neylan, Henn-Haase, & Marmar, 2011). However, continuous exposure to chronic and severe stress and potential traumatic events, associated with the unpredictability of the job, is associated with significant physiological reactivity (Gilmartin, 2002). Enhanced and prolonged physiological stress reactivity can, over time, negatively affect the mental and physical health of officers (Andersen et al., 2010; Violanti et al., 2006). Police officers are at elevated risk of mental health conditions such as depression, posttraumatic stress disorder (PTSD), burnout, and substance abuse (Asmundson & Stapleton, 2008; Austin-Ketch et al., 2012) and elevated risk of physical health conditions such as heart disease, gastrointestinal disorders, and diabetes and even early mortality (Violanti et al., 2005; Violanti, Vena, & Petralia, 1998).

Physical and mental health symptoms can affect occupational performance. Covey and colleagues (2013) found that police officers with symptoms of anxiety were more likely to shoot inappropriately in simulated critical incidents. Furthermore, organizational policies that the officer cannot control, shift work, and family stressors add to the burden of multiple and cumulative operational stressors (Papazoglou, 2013). Ultimately, the cumulative stress, health conditions, and burnout increase the risk of absenteeism, job dissatisfaction, and poor job performance (Conrad & Kellar-Guenther, 2006; Norvell, Belles, & Hills, 1998; Wright & Saylor, 1991). Given the marked risks of cumulative stress on the health and quality of life for the officer, and concern for public safety, researchers have begun to develop resilience-building intervention programs to address stress-related problems for patrol officers (Arnetz, Arble, Backman, Lynch, & Lublin, 2013; Arnetz, Nevedal, Lumley, Backman, & Lublin, 2009; Backman, Arnetz, Levin, & Lublin, 1997). However, resilience interventions have not been applied to SWAT officers. In this article, we review two existing resilience intervention programs designed for patrol officers (Arnetz et al., 2013; Arnetz et al., 2009; Backman et al., 1997; McCraty, Atkinson, Lipsenthal, & Arguelles, 2009, 2012) and detail the manner in which we combined these programs and tested the feasibility of this combined program to address stress responses among SWAT officers.

### Existing Resilience Interventions for Patrol Officers

Empirical evidence is clear that chronic and uncontrollable stressors confer the largest toll on physical and mental health (McEwen, 1998; Sapolsky, 2004). Therefore, prior interventions targeting police stress were based on the premise that providing officers with techniques to reduce the stressfulness of critical incidents and enhance the perception of predictability and control would improve health and performance outcomes (Arnetz et al., 2013; Arnetz et al., 2009; Backman et al., 1997). To test this theory, Backman and colleagues (1997) and Arnetz and colleagues (2013; Arnetz et al., 2009) designed and conducted a 10-week randomized, controlled intervention that contained the following components: (a) psychoeducation regarding stress responses to critical incidents, (b) the use of guided imagery to facilitate officer exposure to critical incident stress while in a safe classroom environment, (c) the application of relaxation techniques to manage stress reactions, (d) mental rehearsal of police best practices (i.e., tactical skills) during the imagery exposure, and (e) learning coping strategies to address the health effects of stress. This intervention strategy was applied to Swedish Police officers as they completed their initial training (Arnetz et al., 2013; Arnetz et al., 2009; Backman et al., 1997). Significant and clinically relevant improvements were found at follow-up, specifically, enhanced problem-based coping skills, reductions in psychological distress, and improvements in physical health (i.e., reduced digestive distress, fewer sleep problems, and less exhaustion).

McCraty and colleagues (2009, 2012) designed and tested a stress reduction intervention based on the premise that reducing psychophysiological reactivity (i.e., cardiovascular and respiratory parameters) during critical incident scenarios would improve health and performance outcomes for police and correction officers in the line of duty (McCraty et al., 2009, 2012). Classroom sessions (approximately 16 hr total) included five training modules covering the following topics: (a) psychoeducation about stress, risks to health, and job performance; (b) instruction on a breathing technique to be used during stressful situations to control physiological responses to stress (e.g., to enhance physical and emotional control during critical incidents); and (c) instruction about communication and coping strategies to be applied in the workplace and at home. Prior to the training program, participants took a number of health measures (i.e., sleep, stress hormones, heart rate variability, c-reactive protein, cholesterol, blood pressure, and glucose regulation) to indicate stress responsivity, risk for cardiovascular disease, and mental health symptoms (i.e., negative mood, depressive and anxiety symptoms, vitality). Researchers found improvements in all health parameters before, during, and following critical incidents and at long-term follow-up (McCraty et al., 2009, 2012). Mental health gains such as increased positive emotion, vitality, reduced negative emotion and depressive symptoms, and improved self-regulation in response to stress were demonstrated (McCraty et al., 2009, 2012). Furthermore, the authors reported significant health savings to police organizations in terms of a 14% reduction in annual health care costs, which translated into a cost savings of $1,179 per employee per year.

Given the significant positive outcomes of both the above-mentioned resilience interventions for patrol officers, we applied a combination of these programs to test the feasibility of resilience programming for SWAT officers. To maximize the benefits of both the Arnetz and colleagues (2013; Arnetz et al., 2009; Backman et al., 1997) and McCraty and colleagues (2009 and McCraty and colleagues (2012) interventions, the psychosocial components of the Arnetz et al. (2013; Arnetz et al., 2009) program were combined with the psychophysiological techniques of the McCraty and colleagues (2009, 2012) intervention.

### The Current Study

Our prior work has demonstrated that, although highly trained, SWAT officers are not immune to the stressors of the job and experience significant stress responses in training exercises and real-world missions (Andersen et al., 2015). We extend prior literature by examining both the psychological and physiological responses to a resilience intervention applied to SWAT officers. We hypothesized that (a) SWAT team officers would be actively engaged in the resilience promotion training intervention delivered to them as part of their regular tactical training program and (b) officers would be better able to control their physiological stress responses, as measured by heart rate and controlled respiration, over the course of the resilience training program as they learned and practiced the program techniques (controlled breathing and mental imagery in response to audio exposure to critical incidents).

## Method

### Participants

Participants (*n* = 18) were male SWAT team police officers (*M* age = 33.66; *SD* age = 4.81; range age = 13) serving in Finland. Although we use the term SWAT for parsimony in this article, the established name of the teams in Finland are known as Special Response Teams (SRTs). There are both regional SRTs and federal SRTs in Finland. The participants in this article consisted of members of regional SRTs. The average number of years of police service (*M*-regular service) was 9.75, and the number of years specifically served in the SWAT team (*M*-SWAT) was 6.67. This study was approved by the University of Toronto as well as the Finnish National Police Research Board. All participants accepted our invitation to participate in the resilience intervention program and signed the ethics approved consent form.

### Procedure

The resilience training program was delivered to participants at the Police University College of Finland located in Tampere, Finland, by licensed psychologists and an experienced SWAT trainer (who was trained in the techniques by the study authors). Participants were completing a 5-day tactical training program that included both classroom and simulated town critical incident scenarios (e.g., local train station, bus and apartment searches and hostage situations). The resilience intervention was conducted in the following manner. On each of the 5 days, a 60-min resilience training session was held, which included the psychoeducational and imagery components of the Arnetz and colleagues (2013; Arnetz et al., 2009) intervention. Specifically, each session began with (a) a 10-min overview of the relevant topics of stress and stress management in policing; (b) the facilitators answered any concerns or questions raised by the participants related to previous sessions’ procedures; (c) the practice of the psychophysiological techniques outlined in the McCraty and colleagues intervention (2009, 2012). Specifically, the psychophysiological intervention consists of focusing on positive emotion while engaging in controlled breathing (5 s inhale and 5 s exhale) arising from the chest (Institute of HeartMath, 2014; McCraty & Atkinson, 2012). (d) While engaging in controlled breathing, the officers listened to critical incident scenarios on iPod devices. The scenarios were developed by Arnetz and colleagues (2013; Arnetz et al., 2009; Backman et al., 1997) and represented police incidents that were rated, by police officers and trainers with many years of experience, as the most stressful incidents that police typically encounter in the line of duty. Officers were instructed to imagine themselves involved in the critical incident and what actions they would take. Scenarios were translated into Finnish and recorded by Finnish actors. Scenarios increased in severity over the course of the 5 days, thus enhancing the potential for increased stress responses: Day 1—car chase, Day 2—armed robbery, Day 3—crazed man, Day 4—domestic violence, Day 5—murder scene. Each scenario had two recordings: (a) a basic scenario that simply described a critical incident from the perspective of the officer and was developed to arouse physiological and emotional responses from the participants and (b) an advanced scenario that was the same as the first but included the voice instructions of the “best practices” from a trained police officer to aid the participant in knowing the correct action to take during such a critical incident. The purpose of listening and imagining oneself in a critical incident while in a controlled, non-stressful environment is to instill confidence and familiarity into the police officer so that when they encounter such a stressor in the line of duty the incident is more predictable, controllable, and thus less threatening (Arnetz et al., 2013; Arnetz et al., 2009). Between the basic and advanced component of each scenario, participants received feedback from the SWAT trainer facilitator. Following the advanced component of the scenario, the facilitators encouraged participants to share their thoughts and feelings related to the session’s imagery and breathing techniques. At the end of each session, the psychologist facilitators debriefed participants. In addition, participants were given 15 extra minutes during the evening to practice the psychophysiological breathing technique.

### Measures

#### Stress reactivity

##### Heart rate

Participants were fitted with a chest-band monitor (i.e., Zephyr Bioharness) to collect heart rate and respiration parameters. The chest-band is non-invasive and fits next to the skin under clothing. This method of measurement has formally been used with police and correctional officers (McCraty & Atkinson, 2012; McCraty et al., 2009). Physiological signals were recorded (moment- by-moment) on the device and were reviewed and analyzed by the researchers at a later date. A person’s heart rate rises as a result of autonomic nervous system activation during a stressful incident (Anderson, Litzenberger, & Plecas, 2002). A moderate, as opposed to extreme, increase in the heart rate during a stressful situation indicates better adaptability and more optimal mental and physical functioning during stress (Anderson et al., 2002; Vrijkotte, van Doornen, & de Geus, 2000). We operationalized improvement in stress response physiology as a participant’s ability to reduce heart rate arousal during the critical incident scenarios, indicating a moderate versus high increase in heart rate over the course of the 5-day intervention, even as the critical incident scenarios became increasingly more stressful. Two measures of heart rate stress reactivity were calculated. First, heart rate max (HR_max_) indicates the highest heart rate reached during the critical incident scenario, and second measure is heart rate average (HR_average_), the average heart rate maintained during the critical incident scenario.

##### Achieving autonomic nervous system control

The psychophysiological technique, as outlined by McCraty and Tomasino (2004), consists of controlled breathing (5 s inhale and 5 s exhale) and has been shown to result in the increased synchronization of the sympathetic and the parasympathetic nervous system. The resulting benefits include (a) reduced stress reactivity and basal level stress hormones (i.e., cortisol) and cardiovascular reactivity (Arnetz et al., 2009; McCraty & Atkinson, 2012), and (b) control of autonomic nervous system activity does not allow for extreme anxiety or panic to occur, but instead the individual is able to maintain a moderate level of arousal, which has been shown to be ideal for logical thought and application of learned knowledge (Jamieson, Mendes, Blackstock & Schmader, 2010). To measure the improvement in autonomic nervous system control, participants wore a pulse oximeter device attached to their earlobe. This device plugged into an iPod with an application (*Inner Balance* for iOS 30 Pin Sensor) for viewing the data in real time as it was collected and saved. The device measures oxygen saturation in the blood calculated from pulse rate, heartbeat, and respiration. The *Inner Balance* software uses visual and auditory biofeedback that trains users how to maintain a certain breathing rate to synchronize sympathetic and the parasympathetic nervous system activity (McCraty & Tomasino, 2004). The *Inner Balance* app provides information about user’s ability to attain a synchronized state (i.e., coherence score) and ability to maintain this state (i.e., achievement score) as measured by the amount of time spent in a coherent state. The Inner Balance application provides a scale of the coherence score level (0.5 *basic–good beginner level*, 1.0 *good*, 2.0 *very good*, 3.0+ *excellent*; Institute of HeartMath, 2014). Similarly, the achievement score refers to all coherence scores calculated every 5 s during a session (Institute of HeartMath, 2014). An achievement score of 300 points per day is associated with stress hormone, cardiovascular and cognitive improvements (Institute of HeartMath, 2014; McCraty & Atkinson, 2012).

### Focus Group—Group Feedback Session

After the completion of the resilience training program, researchers, SWAT team trainers, and SWAT team officers (participants) of the study had a focus group—feedback session scheduled. The first and second authors of this article were the group facilitators of the session. The aim of the focus group was for researchers and SWAT team trainers to listen to the feedback of the participants about the techniques (breathing and imagery) included in the resilience training program. Participants were encouraged by researchers to (a) share their experiences in participating in the resilience promotion program, (b) suggest possible changes about the scenarios presented in the daily sessions, (c) provide feedback about the specific techniques applied over the resilience program, and (d) share with the group any additional general comments or recommendations they had for similar future resilience research endeavors with SWAT teams. At the end of the focus group session, group facilitators administered a brief five-question survey to be completed by the participants. The brief survey was developed on a Likert-type scale (1 = *strongly disagree*, 5 = *strongly agree*) and aimed to receive participant’s feedback on the effectiveness of the training in helping them reduce their stress levels. Some of the questions included in the brief survey were as follows: “Do you believe that this training helped you reduce your stress responses to critical incidents?” “Do you think that the breathing technology used helped you to decrease your stress responses?” “Would you recommend the techniques instructed over the training to be taught to other officers?”

## Results

### Stress Reactivity to Critical Incident Scenarios

#### Cardiovascular stress reactivity

**Figure 1:**
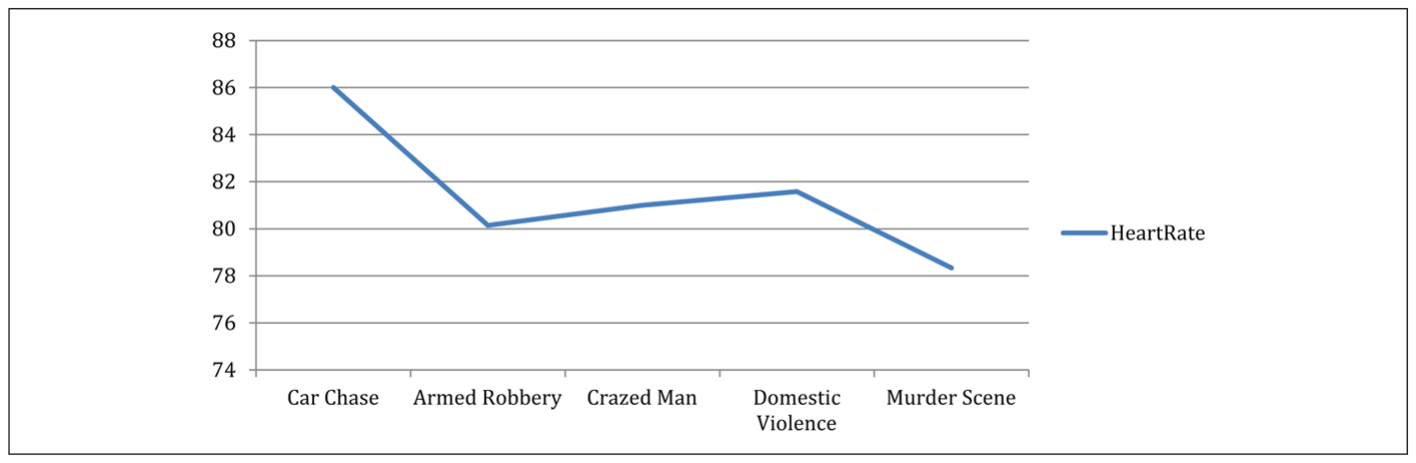
Heart rate mean (HR_mean_) over resilience training.

**Figure 2:**
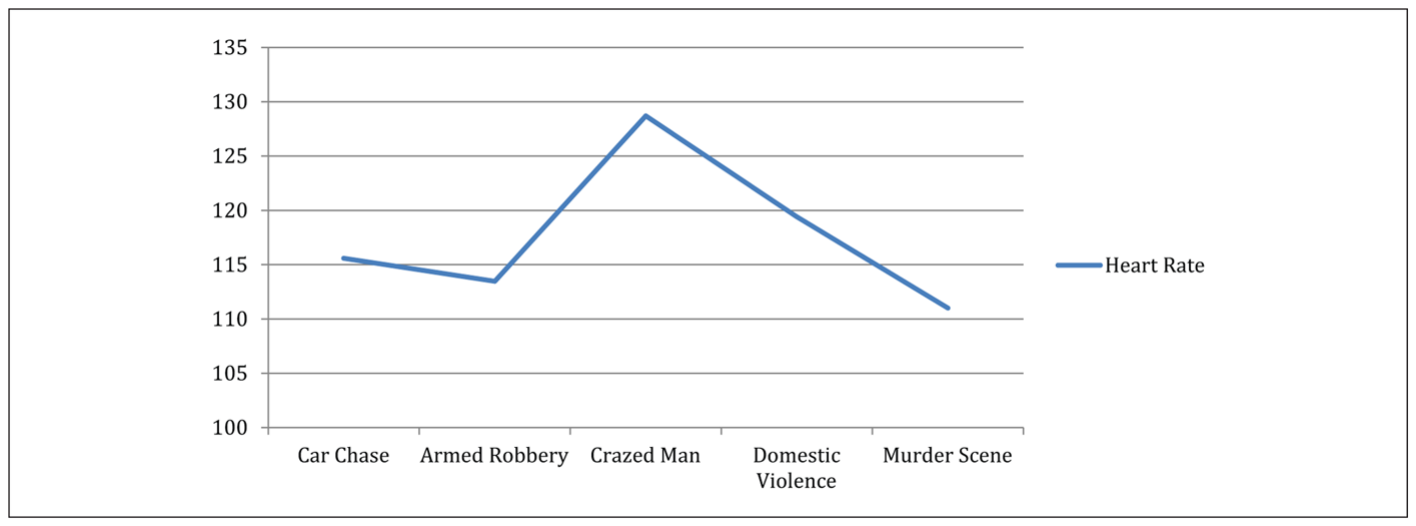
Heart rate maximum (HR_max_) over resilience training.

*T*-test analyses revealed a reduction in average heart rate (HR_mean_) between Day 1 (HR_mean_ = 84; *SD* = 8.28) and Day 5 (HR_mean_ = 78; *SD* = 7.97, *t* = 4.09; *p* < .001) during the period that the SWAT officers were listening to critical incident scenarios (see Figure 1). There were no significant differences in the maximum heart rate observed between Day 1 (HR_max_ = 107; *SD* = 21.18) and Day 5 (HR_max_ = 106; *SD* = 8.48; see Figure 2).

### Autonomic Nervous System Control

#### Controlled breathing

*T*-test analyses revealed an improvement in achievement scores from the beginning of the training (Day 1 = 1.81; *SD* = 0.87) until the end of the training (Day 5 = 1.83; *SD* = 0.59). There were no significant differences in coherence scores observed between the first (Day 1 = 1.81; *SD* = 0.87) and last day (Day 5 = 1.83; *SD* = 0.59).

### Group Feedback

During the focus group session, participants referred to the important role of the resilience promotion training program in helping them better manage stress. Officers also contributed constructive comments about the adaptation of the program for training SWAT teams in the future. For instance, they mentioned that the delivery time of the resilience program was too brief and recommended a program that was longer than five sessions. They also suggested that the scenarios be better tailored to the type of team maneuvers and weapons relevant to SWAT team’s specific tasks. For instance, they noted that they respond to the critical incidents as a group and they never respond to the incidents in dyads.

Results from the brief surveys related to the SWAT team officers’ satisfaction in participating in the training were as follows: Most of the participants (61.11%) were very or highly satisfied with the stress reduction techniques. In terms of the technology used over the training sessions, most participants (72.23%) reported that the devices used were very helpful or highly helpful for them to facilitate their stress reduction processes. Similarly, the majority of the participants (72.22%) agreed or strongly agreed that the technology used over the training is capable of helping individuals manage stress in their daily lives. In terms of knowledge dissemination, participants reported that they were confident (72.22%) or highly confident that they would recommend the techniques of the resilience training program to their peers. Similarly, most of the participants stated that it is important (83.33%) or vitally important to provide this resilience training to their peers.

## Discussion

The aims of this study were to (a) examine if a resilience training program was relevant and accepted by SWAT team officers and (b) assess participants’ physiological stress responses (heart rate, controlled respiration) during the resilience training sessions to note if there were improvements in stress responding over time. In accordance with the first aim, we found that the SWAT officers were eager to participate in the resilience promotion training program and they all completed the program. The officers reported learning, some of them for the first time, about stress management and resilience promotion techniques over the course of the program. The officers reported benefits of the program, including self-reported reductions in stress physiology in reaction to the scenarios and believed this reduction in stress may improve their performance in the line of duty. Participants also reported that they felt that resilience training should be provided to SWAT officers and would recommend the program for their peers.

In accordance with the second aim of the study, our findings revealed that participants were able to significantly reduce their physiological stress responses over the course of training. Specifically, reductions in cardiovascular arousal, as measured by average heart rate, and improved autonomic nervous system control, as measured by the achievement scores on the iPod devices, were observed. Participants significantly improved in their ability to engage in controlled respiration (i.e., breathing; achievement scores) from the first session (Day 1) to the last session (Day 5) of training. The findings from the coherence and achievement scores are in line with what we would expect trained athletes and highly trained police officers to display. Specifically, participants’ coherence scores were high from the beginning of the training (good to very good coherence score = 1.8), not showing a significant improvement over time. This means that SWAT officers were already quickly able to achieve a state of autonomic nervous system control. They understood the technique rapidly and were able to successfully maintain a balance between sympathetic and parasympathetic control while sitting stationary. This finding is not surprising, given that athletes and police trained in marksmanship (both of which apply to SWAT officers) are required to maintain high levels of physical functioning and body control during focused activities. However, what SWAT officers had not been taught before was the ability to maintain and improve autonomic nervous system control *during* highly stressful incidents with potential life threat. A moderate, as opposed to extreme, increase in physiological arousal during stressful situations indicates better adaptability and more optimal mental and physical functioning during stress (Anderson et al., 2002; Vrijkotte et al., 2000). Maintaining moderate arousal is critical to the ability of a SWAT officer to think logically during life and death situations and make decisions in line with their training (Covey et al., 2013). The SWAT officers’ achievement scores reflected their improvement in the ability to achieve physiological control and *maintain* this control even as the critical incidents became more graphic, complex, and stress arousing.

A resilience promotion training program had never been applied to SWAT team police officers before this current study. However, we did not have to “re-invent the wheel” since Arnetz and colleagues (2013; Arnetz et al., 2009)) and Backman and colleagues (1997) successfully applied a resilience promotion training program to police recruits. Therefore, our main goal was to apply Arnetz and colleagues’ (2013; Arnetz et al., 2009) and Backman and colleagues’ (1997) program to SWAT team police officers.

During the focus group session, participants provided both researchers and trainers with constructive feedback based on their experience with the SWAT team. More specifically, they suggested that a resilience training program could be ought to include more than five sessions, as five sessions were considered a brief period of time for the participants to familiarize themselves with the instructed techniques and begin applying these techniques effectively. For instance, Arnetz and colleagues (2013; Arnetz et al., 2009) had 10 (90-min) scheduled sessions for their resilience training with police recruits. In this study, we only had five scheduled (60-min) sessions because the SWAT team’s training program was inundated with numerous other activities (e.g., tactical training, defense tactics, shooting range), and the training was scheduled to be completed within 5 business days. Furthermore, participants suggested that the audio-recorded scenarios needed to be better customized to the SWAT team tasks. For instance, some of the scenarios referred to a police officer and his or her partner as opposed to the team-oriented (all platoon) mission of the SWAT. In addition, other scenarios (e.g., crazy man scenario) described officers patrolling and their conversations with the “crazy man.” Participants in this study reported that the SWAT team’s mission is to respond to a “crazy man” situation and instantly arrest him, so that public safety would not be at risk. It is interesting to note, however, that the largest physiological increases (see Figure 2) were in response to the interaction with an individual with a mental health condition. The findings are in line with the self-reports from both patrol and SWAT officers that, typically, officers face the hardest time addressing mental health situations, for example, when to shoot or not. Also, the consequences of shooting a person with a mental health disorder can be severe. Based on our current findings, we recommend that additional imagery time be spent addressing the concerns and challenges that officers face in managing issues related to mental health and policing. Relevant training is becoming particularly important in these times of increasing terrorist threat. SWAT members have to make quick decisions to place a suspect into a particular category.

### Implications

This study reflects the first attempt to customize a stress resilience intervention to SWAT officers. The intervention was developed from two evidence-based resilience programs for patrol officers. The outcomes of the study indicate that the application of a resilience promotion training program to SWAT officers is feasible, well received, and physiologically beneficial. We include suggestions from SWAT officers to increase the length and specificity of resilience program and training sessions to cover more SWAT-specific incidents and provide greater time for practice of the techniques.

### Future Directions

Results suggest that an intervention to reduce stress responses of SWAT officers to critical incident scenarios works in a simulated training setting. Translation of these findings to real-world occupational hazards is a recommended next step. A randomized controlled trial, testing the efficacy of resilience training among SWAT in real-world settings, is the next step for researchers to pursue.
